# Safety and Efficacy of Phenylephrine Nasal Drops in Bronchiolitis

**Published:** 2014-10-05

**Authors:** Gholamreza Soleimani, Marzieh Akbarpour, Mehdi Mohammadi

**Affiliations:** 1 Research Center for Children and Adolescents; 2 Department of Pediatrics; 3 Department of Epidemiology and Biostatistics, Zahedan University of Medical Sciences , Zahedan , Iran

**Keywords:** Respiratory Tract, Bronchiolitis, Alpha Agonist, Phenylephrine

## Abstract

***Objective:*** Bronchiolitis is a common lower respiratory tract infection in the first year of life. In this disease upper respiratory tract infection is associated with nasal congestion, respiratory distress and hypoxia. We studied the effect of phenylephrine drops as a decongestant in treatment of light and moderately severe cases of acute bronchiolitis.

***Methods:*** This is a double blind randomized trial involving 100 children aged 4 weeks to 12 months. The patients were divided into two groups, the first group received 0.1 ml phenylephrine 0.5% and the second group 0.1 ml sodium chloride (NaCl) 0.9% as placebo in both nostrils. Respiratory rate, heart rate, O2 saturation, dyspnea, retractions and wheezing were assessed before and 30 minutes after medication.

***Findings:*** After medication, O2 saturation and respiratory muscles retractions in the phenylephrine group were significantly better than those of the placebo group (*P*=0.004 and *P*=0.002, respectively). In the phenylephrine group, O2 saturation, retractions and wheezing were also significantly better before than those after medication (*P*=0.003 and *P*<0.0001 respectively). In the placebo group no significant difference before and after intervention was observed.

***Conclusion:*** Phenylephrine as a topical decongestant is an inexpensive, easily available and suitable means in the treatment of mild to moderately severe bronchiolitis.

## Introduction

Acute bronchiolitis is the most common lower respiratory tract infection in the first year of life and also the most common reason for the infants’ hospitalization during winter season^[^^1^^,^^2^^]^, respiratory syncytial virus being the most common cause. Unfortunately no remarkable progress in its treatment is achieved in recent years^[^^3^^]^.

 Involvement of upper airways in bronchiolitis leads to nasal congestion, inadequate oral intake, dehydration, respiratory distress and hypoxemia ^[^^2^^]^. Therefore, nasal decongestants may relieve the signs and symptoms related to upper airway obstruction. Phenylephrine as an alpha-agonist is supposed to reduce nasal edema^[^^4^^]^.

 The effect of epinephrine^[^^5^^-^^7^^]^, inhaled furosemide^[^^1^^]^, inhaled hypertonic saline^[^^8^^-^^10^^]^, xylomethazoline^[^^2^^]^, phenylephrine^[^^4^^]^, dexamethasone ^[^^11^^]^ and zinc sulfate^[^^12^^]^ has been investigated in treatment of bronchiolitis. Nevertheless, the only non-controversial treatment remains supportive therapy^[^^3^^]^.

 The results of studies on the effect of α-blockers as decongestant are controversial. Therefore, we tried to compare the effect of intranasal phenylephrine (as a decongestant) with sodium chloride (NaCl) 0.9% (as placebo).

## Subjects and Methods

This double blind randomized clinical study was conducted in *Ali-*Ebne*-Abitaleb* hospital, Zahedan, Southeast Iran. All patients aged 4 weeks to 12 months with clinical diagnosis of viral bronchiolitis were eligible for the study. Clinical diagnosis of viral bronchiolitis was based on the first episodes of wheezing in infants with a viral upper respiratory tract infection^[^^13^^]^. Wheezing was evaluated with a stethoscope and defined as end-expiratory or expiratory according to the physician’s hearing. Oxygen therapy was defined as oxygen supply via head box to increase blood O_2_ saturation to more than 90 percent.

 Patients with following criteria were excluded: gestational age less than 34 weeks; heart rate more than 200 beats/min; respiratory rate >70 breaths/min; use of α- or β-agonists during 24 hours before admission; hypotension; chronic disease, history of atopia in first degree relatives, known cases of cystic fibrosis and any signs and symptoms of severe bronchiolitis.

 The medical staff involved in the study was blinded. Both drops were smell- and colorless and just labeled A and B. Based on permuted-block randomization, patients were allocated to receive phenylephrine 0.5% drop (A) or NaCl 0.9% drop (B) in both nostrils while in supine position and remained in this position for one minute.

 Case and control group both received routine treatment for bronchiolitis (O_2_ therapy, salbutamol spray, nasal suction and neubulizer). Case group received in addition phenylephrine 0.5%, one drop in each nostril, which was replaced by NaCl 0.9% in the control group.

 O_2_ saturation, respiratory rate, heart rate, retractions, dyspenea and wheezing were evaluated by the same investigator thirty minutes after administration of the drug and placebo.

 Dyspenea was defined as one or more of the following items: difficulty in feeding; decreased vocalization, and/or agitation. Retractions were noted as no retractions, intercostal retractions or 

subcostal+inter-costal retractions.

 To determine the sample size, mean respiratory rate was taken to be 43 in treatment and 42 in control group with 1.6 as standard deviation^[^^4^^]^, 5% and 20% type I and II error respectively. Therefore sample size was calculated to be 50 patients in each group.

 Analyses of data were done by the SPSS (version 16) using chi-square, Wilcoxon, Kruskal-Wallis and independent sample t-test. The level of significance was considered according to 95% confidence interval. 

## Findings

One hundred and seven patients (38% boys) between 4 weeks and 1 year old (mean age 6.02±3.2 months) were eligible to participate in the study. Seven patients were excluded because of prematurity (2 cases), unstable vital signs (2 cases), severe bronchiolitis (2 cases), or congenital heart disease (1 case). 

 In the initial examination there was no significant difference between the two groups in demographic data including heart rate, respiratory rate, O_2_ saturation, retrac-tions, wheezing, and dyspnea (Table 1).

 Thirty minutes after administration of phenylephrine drops, O_2_ saturation significantly increased in group A (*P*=0.004) whereas the severity of retractions significantly decreased (Table 2).

 Comparison of data showed that O_2_ saturation significantly increased in the case group after phenylephrine drops (*P*=0.003) and the severity of retractions and wheezing significantly decreased (*P*<0.0001 and *P*=0.006 respectively) (Table 3).

 There were no statistical differences in any of the outcome measures in group B before and after medication (Table 4). No adverse effect was seen after application of the decongestant.

## Discussion

This study demonstrated that phenylephrine (as a nasal decongestant) is more effective than normal saline solution (as a placebo) to treat acute bronchiolitis and reduce clinical severity of the symptoms.

**Table 1:** Demographic characteristics and pre-treatment data

**Parameter**		**Phenylephrine (n=50)**	**NaCl (n=50)**	***P*** **. value**
**Age **		6.6 (3.4)α	5.4 (3.04)α	0.08
**Sex**	Male Female	21 (42%) 29 (58%)	17 (34%) 33 (66%)	0.4
**Retraction**	No retractions Intercostal Subcostal + intercostal	5 (10%) 19 (38%) 26 (52%)	1 (2%) 23 (46%) 26 (52%)	0.7
**Dyspnea**	NL feeding, vocalization and activity One of the following: difficulty in feeding; decreased vocalization; or agitation Two of the following: difficulty in feeding, decreased vocalization, and/or agitation	5 (10%) 25 (50%) 20 (40%)	7 (14%) 14 (28%) 29 (58%)	0.2
**Wheeze**	End-expiratory Expiratory	14 (28%) 36 (72%)	17 (34%) 33 (66%)	0.5
**O**_2_ ** saturation**		93.1 (4.2)α	92.1 (4.03)α	0.2
**Respiratory rate**		44.7 (9.3)α	47.5 (9.5)α	0.1
**Heart rate**		115.3 (9.9)α	118.2 (8.7)α	0.1

α : Mean (Standard Deviation); NaCl: Sodium Chloride

Alpha-adrenergics produce vasoconstriction and decrease blood flow through microvesseles, leading to decreased resistance to airflow by reducing hyperemia, mucosa swelling, plasma exudation and nasal secretions^[^^13^^]^. In allergic rhinitis, oral and topical decongestants cause vasoconstriction and oppose vasodilation in the nasal mucosa, thus decreasing inflammation and reducing nasal airway resistance so that the nasal congestion is diminished and nose breathing faci-litated^[^^14^^,^^15^^]^.

 There is a wide-range of practice in the management of bronchiolitis^[^^16^^]^ but there are limited studies available on the effect of nasal decongestants in the treatment of pediatric bronchiolitis^[^^2^^,^^4^^]^. According to American Academy of Pediatrics a consistent benefit from α-adrenergic or β-adrenergic agents for bronchiolitis treatment is controversial. So, α-adrenergic or β-adrenergic medication is an option to be more investigated^[^^17^^]^. In a study, nasal decongestant (xylometazoline) was as effective as epinephrine in the treatment of acute bronchiolitis, so it was concluded that upper respiratory tract diseases have an important effect on clinical presentation and pathogenesis of bronchiolitis^[^^2^^]^. Another study assessed the effect of nasal phenyephrine in infants hospitalized for bronchiolitis and concluded that respiratory status does not change after short term use of topical nasal phenylephrine.

**Table 2:** Comparison of data between phenylephrine and placebo 30 minutes after application

**Parameter**		**Phenylephrine (n=16)**	**NaCl (n=16)**	***P. *** **value**
**Retraction**	No retractions Intercostal Subcostal + intercostal	8 (16%) 29 (58%) 13 (26%)	0 (0%) 25 (50%) 25 (50%)	0.002
**Dyspnea**	NL feeding, vocalization and activity One of the following: difficulty in feeding; decreased vocalization; or agitation Two of the following; difficulty in feeding; decreased vocalization; and/or agitation	4 (8%) 30 (60%) 16 (32%)	8 (16%) 13 (26%) 29 (58%)	0.2
**Wheeze**	No wheeze End-expiratory Expiratory	3 (6%) 18 (36%) 29 (58%)	0 (0%) 19 (38%) 31 (62%)	0.5
**O**_2_ ** saturation**		94.1 (3.7)α	91.9 (3.5)α	0.004
**Respiratory rate**		43.9 (9.1)α	47.2 (9.5)α	0.1
**Heart rate**		114.3 (9.7)α	118.1 (10.7)α	0.06

α : Mean (Standard Deviation); NaCl: Sodium Chloride

**Table 3:** Comparison of data before and 30 minutes after phenylephrine application

**Parameter**			**Before (n=50)**	**After (n=50)**	***P. *** **value**
**Retraction**	No retraction Intercostal Subcostal+intercostal	5 (10%) 19 (83%) 26 (52%)		8 (16%) 29 (58%) 13 (26%)	<0.0001
**Dyspnea**	NL feeding, vocalization and activity One of the following: difficulty in feeding; decreased vocalization; or agitation Two of the following: difficulty in feeding; decreased vocalization; and/or agitation	5 (10%) 25 (50%) 20 (40%)		4 (8%) 30 (60%) 16 (32%)	0.3
**Wheeze**	No wheeze End-expiratory Expiratory	0 (0%) 4 (28%) 36 (72%)		3 (6%) 18 (36%) 29 (58%)	0.006
**O**_2_ ** saturation**		93.1 (4.2)α		94.1 (3.7)α	0.003
**Respiratory rate**		44.7 (9.3)α		43.9 (9.09)α	0.07
**Heart rate**		115.3 (9.9)α		114.3 (9.7)α	0.2

α : Mean (Standard Deviation); NaCl: Sodium Chloride

This seemed to be attributed to strict inclusion and exclusion criteria, parental refusal and lack of study personnel on weekends and nights^[^^4^^]^. Diagnostic tests such as radiography and viral antigen test can be applied to confirm diagnosis, although clinical diagnosis is decisive^[^^18^^,^^19^^]^. Therefore our patients were diagnosed clinically. In accordance with previous studies^[^^2^^,^^4^^]^, we observed no adverse effects of phenylephrine as a topical nasal decongestant and found it safe in short course usage. Rebound nasal congestion does not appear after a single dose but it might be seen after recurrent doses^[^^4^^]^.

 Short time assessment of decongestant effect can be seen as a limitation of our study. More studies with frequent usage of the decongestant are needed to determine suitable dosage, duration of therapy, and long term follow up to validate systemic and topical effect and eventual side effects (such as rebound) of the lhenylephrine nasal drops more exactly.

**Table 4:** Comparison of data before and 30 minutes after normal NaCl application

**Parameter**		**Before (n=50)**	**After (n=50)**	***P*** **-value**
**Retraction**	No retractions Intercostal Subcostal & intercostal	1 (2%) 23 (46%) 26 (52%)	0 (0%) 25 (50%) 25 (50%)	1
**Dyspnea**	NL feeding, vocalization and activity One of the following: difficulty in feeding; decreased vocalization, or agitation Two of the following: difficulty in feeding, decreased vocalization, and/or agitation	7 (14%) 14 (28%) 29 (58%)	8 (16%) 13 (26%) 29 (58%)	0. 65
**Wheeze**	No wheeze End-expiratory Expiratory	0 (0%) 17 (34%) 33 (66%)	0 (0%) 19 (38%) 31 (62%)	0.15
**O**_2_ ** saturation**		92.1 (4.03)α	91.9 (3.5)α	0.57
**Respiratory rate**		47.5 (9.5)α	47.2 (9.5)α	0.17
**Heart rate**		118.2 (8.7)α	118.1 (10.7)α	0.95

α ^: Mean (Standard Deviation); NaCl: Sodium Chloride^

## Conclusion

Intranasal phenylephrine as an alpha-adrenergic decongestant, in combi-nation with supportive therapy in bronchiolitis, is a safe, not expensive and effective treatment for mild to moderate viral bronchiolitis for both inpatient and outpatient treatment. Further studies are needed to determine the optimal dosing and time interval to identify the maximum effect.
